# The Proposed 48-Month Emergency Medicine Residency Requirement Demands Immediate Scrutiny

**DOI:** 10.5811/westjem.48551

**Published:** 2025-06-24

**Authors:** Shahram Lotfipour, Ian Olliffe, Stephen Hayden, Soheil Saadat, Mark I. Langdorf

**Affiliations:** * University of California, Irvine, Department of Emergency Medicine, Irvine, California; † Eisenhower Health, Department of Emergency Medicine, Rancho Mirage, California; ‡ University of California, San Diego, Department of Emergency Medicine, San Diego, California

## Abstract

The Accreditation Council for Graduate Medical Education’s (ACGME) proposal to mandate 48-month training for all emergency medicine residency programs represents a significant departure from the current system where both 36- and 48-month formats successfully coexist.

The ACGME’s justification relies on a methodologically flawed survey that never directly asked program directors about optimal training duration. Instead, it calculated totals by summing individual rotation estimates without considering integrated curricula or practical constraints. Even if these results were to be accepted, directors of three-year programs reported a mean desired duration of only 41.6 months—hardly justifying a universal 48-month mandate.

Current evidence contradicts the ACGME’s rationale. Three-year graduates achieve higher board pass rates (93.1% vs 90.8%) and demonstrate equivalent clinical performance to four-year graduates. The mandate would impose substantial financial burdens on trainees—an opportunity cost exceeding $200,000-$250,000—while potentially deterring qualified applicants and discouraging fellowship training.

We urge the ACGME to pause implementation and provide compelling evidence that a 48-month mandate is necessary and demonstrably superior to the current model.

The Accreditation Council for Graduate Medical Education (ACGME) recently proposed a radical revision to the Program Requirements for Graduate Medical Education in Emergency Medicine, mandating a standardized 48-month training duration for all residency programs, which would take effect in 2027.^1^ This is a substantial departure from the current structure, where both 36-month and 48-month formats coexist. As we have previously argued, the value proposition of a fourth-year of training has historically lacked definitive supporting evidence.^2^–^3^ Roughly three-quarters of emergency medicine (EM) residency programs perform successfully under the 36-month (postgraduate years [PGY] 1–3) model, yielding competent physicians who meet the demands of our challenging field.^4^ While we all share the ACGME’s goals of improving resident education and ensuring preparedness for independent practice, imposing a universal 48-month mandate appears premature and insufficiently justified by convincing evidence. The ACGME should not move forward with a mandate of this magnitude—one carrying significant potential drawbacks—without first providing clear evidence that it is both necessary and demonstrably superior to the current three-year training model.

This call for an evidence-based justification before such a significant change is implemented is echoed by major specialty organizations. The Emergency Medicine Residents’ Association (EMRA) has urged the withdrawal of the proposed requirements, advocating for the continued accreditation of both three- and four-year programs based on evidence.^5^ Similarly, the Society for Academic Emergency Medicine, representing a broad constituency including academic chairs and clerkship directors, has expressed concerns about the accelerated timeline and lack of consensus, requesting a pause in the implementation process to allow for more thorough consideration and stakeholder engagement.^6^

The ACGME justifies this proposal by citing the need to accommodate an expanded curriculum.^1^ The council identified this need through a survey administered to program directors (PD) by members of a designated Program Requirements Writing Group (PRWG), who designed the survey based on takeaways from their summit with stakeholders. A closer look at this process reveals many concerns with their methodology and resulting conclusions. The PRWG was comprised of eight emergency physicians, all previously or currently serving on the ACGME Review Committee for Emergency Medicine.^7^ These eight physicians notably constituted 25% of the total “stakeholders” who joined the summit.^7^ There is also a fundamental lack of detail describing specific expertise to opine on the educational domains involved across both the PRWG and the other stakeholders involved with the summit. The ACGME’s over-representation in the summit could have introduced bias and excessively influenced the “consensus” on which it decided to build the ideal curriculum.^7^ This is even more striking given the lack of clarity regarding the stakeholders’ qualifications to influence such a consequential survey.

The survey itself suffers from methodological and interpretative issues. It did not directly ask PDs what they believed to be the optimal total program duration for EM residency. Consequently, the survey results do not necessarily and accurately represent respondents’ views on the total duration required. Instead, the PRWG calculated a total by summing up PDs’ estimates of the time needed for individual curriculum components derived from their summit, arguing that this approach avoided bias from current program formats.^7^ The PDs were also explicitly asked to estimate the required time for these rotations “without considering current ACGME EM training requirements, your current curriculum, or your current program resources” to achieve “autonomous practice.” By instructing PDs to estimate rotation durations without considering the broader context of that rotation within current training requirements, individual program curricula, or institutional resources, the survey restricted their ability to account for integrated curricular design. Specifically, it did not permit consideration of how multiple educational objectives might be addressed concurrently through combined rotations or interdisciplinary experiences.

Such integration has the potential to reduce the total number of required rotations while still achieving equivalent educational outcomes. Furthermore, this framing is based on idealistic rather than strictly essential criteria to achieve competency for autonomous practice, which could have significantly inflated the perceived time needed for the total program length. These questions also lacked any time constraint, allowing respondents to suggest unlimited durations for each rotation; the sum of these individual suggestions could easily exceed a practical or intended total program length. It would be critical to know the range of total suggested durations and analyze separately those responses that, when aggregated, would exceed a 48-month program. A more effective methodological approach might have been to individually inquire about appropriate durations for each of the four curricular sections and then follow up with the individual rotations. The median suggested duration for each section could then inform an agreement on overall residency duration.

Furthermore, the interpretation of these suggested durations must consider external variables. The respondents’ suggested durations for rotations likely reflected their own institutions’ patient loads, emergency department (ED) environment, and faculty experience. For example, PDs from high-volume institutions might propose shorter rotation durations due to an anticipation of sufficient patient exposure. In contrast, those from low-volume institutions may suggest longer durations to achieve the same. This variability needs to be accounted for when analyzing the survey results.

Even when taking these results at face value, the ACGME cites them unreliably. They point to a mean desired training length of approximately 43.4 months. This figure, however, is skewed by responses from existing four-year programs, whose directors desired over 50 months. This overall “average” overshadows that directors of three-year programs reported a mean of 41.6 months, which hardly indicates a universal demand for a 48-month mandate. Additionally, while the survey instrument is available as a supplement in the PRWG’s complete publication, the significant details regarding question framing, the lack of direct inquiry about total program length, and the absence of analysis of confounding variables or excessively long cumulative durations are notably absent from the ACGME’s proposed revisions to program requirements.

While surveys have value, a significant residency program change should ideally be informed by objective measures and more robust evidence. Given that both three- and four-year EM residency programs exist, evaluating their respective effectiveness using objective performance metrics would provide more reliable guidance. The appropriate residency duration for clinical experience primarily relates to case exposure stratified by patient type, with duration as only a surrogate measure. Therefore, the survey’s conclusions supporting the mandate are derived from surrogate variables, which is not the optimal method when the possibility of directly comparing the performance outcomes of graduates from three- vs four-year programs exists.

The ACGME avoids this direct comparison in their proposal even beyond their concerns about curriculum. For example, they point to shortened shifts leading to decreased patient encounters. While this merits attention, increasing the required weeks in the ED is an indirect response; it does not guarantee reaching the desired quantity or quality of patient encounters.^8^ The actual educational yield is heavily influenced by factors unrelated to program length, including patient volume and acuity, hospital boarding, the roles of non-physician practitioners, and individual resident experience and efficiency.^8^–^11^ A more direct and potentially more effective method, as advised by the EMRA, involves establishing minimum encounter targets, such as 5,000–6,000 total patient encounters, including specific targets for pediatric and critical care patients, that could demonstrably be met within a well-structured three-year program (eg, 94 ED weeks).^8^

Another troubling rationale involves the reference to declining American Board of Emergency Medicine (ABEM) pass rates.^1^ While the ACGME acknowledges the general trend,^12^ they have not presented comparative evidence showing that graduates from four-year programs achieve better results on these examinations than their three-year counterparts.^8^ In fact, current data seems to indicate the opposite. A 2023 ABEM analysis of 2018–2020 outcomes found that three-year EM graduates achieved a statistically significant higher pass rate on the Qualifying Examination than four-year graduates (93.1% vs 90.8%).^8^, ^13^ For the Oral Certification Examination, pass rates showed no statistically significant difference between the groups; the minor variations observed are unlikely to represent a meaningful difference in educational outcomes.^14^

Furthermore, recent studies evaluating early-career clinical performance have not identified an advantage for graduates of four-year programs. An extensive study by US Acute Care Solutions (USACS) detected no significant variations in performance indicators such as patients per hour, relative value units per hour, or 72-hour returns needing admission/transfer, between new third-year and four-year program graduates in their first year.^14^–^15^ The USACS researchers concluded that overall performance concerning “efficiency, safety, and flow were largely similar” between the cohorts.^8^, ^15^ Therefore, using the overall decline in board pass rates to justify mandating a fourth year appears inconsistent with the specific comparative evidence available.^8^ The ACGME needs to provide straightforward data demonstrating the necessity of this mandate.

Mandating a fourth residency year would impose substantial financial burdens on trainees and potentially negatively impact EM recruitment. Comparing an average attending compensation of $385,554 and a PGY-4 salary ≈ $70,000 introduces a significant opportunity cost of at least $200,000-$250,000 and potentially over $300,000 for an additional year at a lower resident salary.^16^–^17^ This compounds existing average medical student debt exceeding $200,000.^18^ The increased time and financial commitment would likely reduce EM’s competitiveness relative to three-year specialties like internal medicine or pediatrics, which is notable given program length’s impact on medical student specialty interest.^19^ This risks deterring highly qualified applicants, which appears unwise during a volatile match period for the specialty.^20^ Furthermore, a mandatory fourth year could discourage graduates from pursuing fellowship training or academic careers. After a longer residency, factors such as the debt burden, burnout from training, and a desire to finally begin full practice may make the prospect of an additional one to two years of fellowship less appealing.^21^–^22^ The proposal imposes unnecessary burden and could shrink the pipeline of future subspecialists and academic leaders.

The ACGME’s proposal to mandate a 48-month training duration for all EM residency programs is a solution in search of a problem that has yet to be defined by compelling evidence. Their justifications for this mandate do not withstand scrutiny based on currently available data. It also imposes significant financial burdens on trainees and carries substantial risks of negatively impacting specialty attractiveness and the pursuit of fellowship training. We urge the ACGME to pause the implementation of this requirement and engage in a transparent process that presents complete data demonstrating why a universal 48-month mandate is necessary and how it will lead to measurable improvements in educational outcomes and patient care that outweigh its considerable costs. The future of EM training deserves decisions grounded in evidence, not assumption. Preserving flexibility and choice in program length, a structure under which most of our specialty currently trains successfully, should remain the standard unless irrefutable evidence proves otherwise. Major reforms in graduate medical education must adhere to the same evidence-based principles we demand in our clinical practice.

Link to Addtional Expert Commentary: https://escholarship.org/uc/item/4zb183gj
